# Temperature-Controlled Chain Dynamics in Polyimide Doped with CoCl_2_ Probed Using Dynamic Mechanical Analysis

**DOI:** 10.3390/ma17030753

**Published:** 2024-02-04

**Authors:** Daniela Ionita, Mariana Cristea, Ion Sava, Maria-Cristina Popescu, Marius Dobromir, Bogdan C. Simionescu

**Affiliations:** 1 “Petru Poni” Institute of Macromolecular Chemistry, Aleea Grigore Ghica Voda 41A, 700487 Iasi, Romania; ionita.daniela@icmpp.ro (D.I.); isava@icmpp.ro (I.S.); cpopescu@icmpp.ro (M.-C.P.); bcsimion@icmpp.ro (B.C.S.); 2 Department of Exact and Natural Sciences, Institute of Interdisciplinary Research, “Alexandru Ioan Cuza” University of Iasi, Blvd. Carol I 11, 700506 Iasi, Romania; marius.dobromir@uaic.ro

**Keywords:** polyimide, thermal imidization, dynamic mechanical analysis, cobalt chloride, viscoelastic behavior

## Abstract

Cobalt(II) chloride (CoCl_2_) being in the vicinity of polyimide chains entails modifications in terms of the molecular dynamics, which are mainly governed by the possible presence of amic acid residual groups, by the transition-metal-type characteristics of cobalt and by the CoCl_2_ content. Polyimide was synthesized using poly(amic acid) according to the reaction of 2,2′-bis(3,4-dicarboxylphenyl)hexafluoropropane dianhydride (6FDA) with 3,3′-dimethyl-4,4′-diaminodiphenylmethane (MMDA) in N,N-dimethylacetamide. CoCl_2_ was added before the thermal imidization of the poly(amic acid). An experimental approach was designed to establish the interaction between the polyimide and CoCl_2_ and whether the interaction depends on the quantity of the salt. Evidence for the existence of residual amic acid groups was obtained using second derivative Fourier Transform Infrared Spectroscopy (FTIR) and with the help of 2D correlation spectroscopy (2D-COS). Moreover, FTIR, along with X-ray photoelectron spectroscopy (XPS), revealed the interaction between the polymer and CoCl_2_, primarily in the form of Co(II)-N coordinated bonds. Nevertheless, the coordination of cobalt with suitable atoms from the amic acid groups is not precluded. The results of dynamic mechanical analysis (DMA) featured a specific relaxation assigned to the presence of CoCl_2_ in the polymeric film and demonstrated that its (non)reinforcing effect depends on its content in the polyimide.

## 1. Introduction

Polyimides are considered to have one of the longest histories in the field of thermostable polymers. Since they were first obtained in the shape of films, using a poly(amic acid) precursor in the laboratories of DuPont, the applications of this class of polymers have been extended due to joining outstanding thermal behavior with excellent mechanical properties in the same materials [1,2,3]. For example, superior film-forming properties have opened up various possibilities for using them in various fields, like microelectronics, coatings and membranes [4,5]. The combination of polyimides and various additives takes advantage of the properties that come from both components [6,7]. As an example, metal salts can be incorporated into polyimide films to make them conductive or magnetic or to impart other properties [8,9,10,11,12,13,14]. For instance, cobalt(II) chloride (CoCl_2_) has been largely used as a dopant to prepare conductive polymers [15,16], as a catalyst for polymerization or pyrolysis [17,18], as a hydrogen storage material [19] and in polymeric films with hexagonally ordered pores [20]. The last two applications rely on the hygroscopicity of CoCl_2_ given that it interacts even with traces of water. Moreover, in the presence of water, CoCl_2_ converts from its pink color, characteristic of the anhydrous form, into blue. The process is reversible under the action of heat or a vacuum [21,22]. Chromism was employed in the construction of optical humidity sensors by including CoCl_2_ in polymers [23,24,25,26]. Furthermore, the presence of CoCl_2_ additives [27,28,29] changes the immediate environment of polymer chains, and this is reflected in their molecular dynamics. The fact that the salt contains a transition metal with incompletely filled d-electronic orbitals should not be neglected. Cobalt ions are able to coordinate with the lone pairs of specific atoms (oxygen or nitrogen) that are part of the structure of the polymer [30]. When this interaction occurs, they can exert their influence on certain decisive properties of the final material. This kind of interaction has been evidenced in CoCl_2_–chitosan films, CoCl_2_–polyvinylpyrrolidone/poly(vinyl alcohol) blends and CoCl_2_–poly(ethylene oxide) hybrid materials, mainly using Fourier Transform Infrared Spectroscopy (FTIR) and X-ray photoelectron spectroscopy (XPS) [25,26,31]. In N-picolyl polyurethane, the transition metal ions act as a crosslinker between two pendent pyridine groups [32]. Huang et al. have used CoCl_2_ as a coordination crosslinker (non-covalent) in acrylonitrile–butadiene rubber. The results of dynamic mechanical analysis (a higher storage modulus on the rubbery plateau) indicated a slight coordination reaction between the cobalt ions and the nitrile groups of the rubber [33].

Polyimide films are usually obtained using the classic two-step method: the synthesis of poly(amic acid) from diamines and dianhydrides, followed by its thermal conversion into polyimide [1,34]. The incorporation of the metal salt into the solution of poly(amic acid) is performed before the thermal cyclodehydration.

Bogges and Taylor have investigated polyimides doped with CoCl_2_, derived from the diamine 4,4′-oxydianiline (ODA) or 4,4′-diaminodiphenylsulfide (DDS) and the dianhydride 3,3′,4,4′-benzophenonetetracarboxylic dianhydride (BTDA) or 4,4′-bis(3,4-dicarboxyphenoxy)diphenylsulfide dianhydride (BDSDA) [35]. They have deduced using XPS and UV–Vis spectroscopy that there is little interaction of the polyamic acids or polyimides with CoCl_2_. Also, Varma et al. have reported, based on IR data, the absence of interactions between polypyromellitimide and CoCl_2_. Nevertheless, the dopant had an influence on the cyclodehydration reaction, and the tensile strength of the polyimide was reduced in its presence [36]. Through investigation of the same polyimide for the purpose of creating a moisture indicator, Halper and Villahermosa have assumed, by taking into consideration an IR analysis, that CoCl_2_ did not inhibit imidization [37]. The studies based on IR spectra rely on checking whether the positions of characteristic bands are changed by the additive. Extensive research had shown that IR measurements are not always sufficient to ascertain the completeness of the imidization reaction or the absence of the interaction of the polymer with the additive. For example, in the range between 1650 cm^−1^ and 1780 cm^−1^, the IR absorption signals of various groups of atoms within the polyimide and the poly(amic acid) are often difficult to separate [38,39,40,41]. The symmetric stretching IR peaks of C=O in imide and the carboxylic group in amic acid are about 15 cm^−1^ apart (around 1730 cm^−1^ and 1715 cm^−1^, respectively), and their individual contributions cannot be quantitatively measured. The amide I band near 1665 cm^−1^ appears less than 100 cm^−1^ apart from them. All of these bands may shift to lower wavenumbers when N–H and C=O groups are involved in hydrogen bonds [42,43,44,45]. Moreover, the picture becomes even more complicated in the presence of CoCl_2_ given that it exhibits vibration bands near 1600 cm^−1^ and in the N–H and O–H stretching vibration region (3100–3500 cm^−1^) [20,26,46]. Because of these complications, other experimental characterizations are often needed in addition to IR measurements. The presence of residual amic acid groups in a polyimide can be easily identified, using dynamic mechanical analysis (DMA), as a supplementary relaxation peak before the main relaxation of the polyimide [47,48,49,50]. Practically, the imidization of incompletely transformed polyimides is continued in the oven of the DMA device. This issue was detailed by a few authors in previous publications [51,52]. There are also instances where imidization was enhanced in the presence of the additive [53,54,55].

Some questions related to the overlap between characteristic bands in the IR spectra have been partially answered with the help of the two-dimensional correlation spectroscopy technique developed by Noda. Basically, by applying a cross-correlation analysis to sinusoidally perturbed IR linear dichroism spectra, the 2D correlation spectra were obtained [56]. Generalized two-dimensional correlation spectroscopy (2D-COS) allows us to apply this analysis technique to the investigation of any reasonable spectroscopic signals (IR, NMR, X-ray, chromatography, etc.) that have been perturbed by any physical or chemical variable (time, temperature, pressure, concentration) [57,58,59]. The 2D-COS method has also been used to analyze the sequences of chemical structure changes during thermal imidization [60,61,62,63,64].

In this work, three perspectives are offered to shed light on the nature of the interaction between CoCl_2_ and the polyimide obtained from 2,2′-bis(3,4-dicarboxylphenyl) hexafluoropropane dianhydride and 3,3′-dimethyl-4,4′-diaminodiphenylmethane. Previous studies have investigated the mechanism responsible for the color change as a function of humidity, as well as the physico-chemical behavior of CoCl_2_-filled fluorinated polyimide materials [65,66]. The use of 2D-COS and the exploitation of the second derivative of conventional FTIR can establish the completeness of the thermal conversion of poly(amic acid) into polyimide. The representation of the vibrational structure designates whether CoCl_2_ acts as more than an additive. The electronic state of the cobalt, determined using the XPS technique, will be indicative of the interaction between the salt and the polyimide, if there is one. These findings will support the assignment of the relaxations revealed in the dynamic mechanical analysis investigations, performed both in the isochronal and multi-frequency modes.

## 2. Materials and Methods

### 2.1. Materials

The cobalt(II) chloride hexahydrate and N,N-dimethylacetamide (DMAc) were purchased from Sigma-Aldrich (St. Louis, MO, USA) and used as received. The 2,2′-bis(3,4-dicarboxylphenyl)hexafluoropropane dianhydride (6FDA) was acquired from the former Hoechst Celanese (Summit, NJ, USA) and purified via recrystallization from acetic acid anhydride. The diamine used in this study, 3,3′-dimethyl-4,4′-diaminodiphenylmethane (MMDA), was synthesized according to a reported method [34].

### 2.2. Synthesis of the CoCl_2_/Polyimide Film Materials

**Scheme 1:**
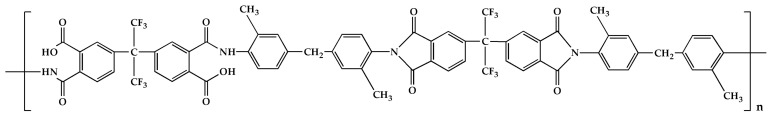
The structure of the polyimide.

The polymer precursor, polyamic acid (PAA), was synthesized according to the reaction of dianhydride and diamine in DMAc, with a 15% solid concentration, using a method previously described [65,66]. According to typical synthesis, 4,4′-diamino-3,3′-dimethyl-diphenylmethane was dissolved in DMAc, and an equimolar amount of hexafluoroisopropylidenediphthalic dianhydride was added under stirring, which was continued for 4 h at room temperature, resulting in a viscous polyamic acid yellow solution being generated. A part of this solution was processed to obtain a pristine polyimide film, and the other part was mixed with different quantities of cobalt chloride. The precursor film was obtained by casting this homogeneous solution onto glass plates and drying it at 100 °C–110 °C for 4 h to evaporate the solvent. The subsequent heating of the precursor films at 150, 180, 210, 240–250, and 260–280 °C consecutively (for 40 min at each temperature) resulted in the final polyimide films. The color of polyimide films that contain CoCl_2_ shifts from bright to dark green, as the content of the salt increases from 0 to 20%: PI, PICo5, PICo10, PICo15, PICo20. The structure of the polyimide films is shown in Scheme 1.

### 2.3. Characterization

The FTIR spectra of the studied samples were measured at a 4 cm^−1^ resolution and using 64 scans on a Bruker ALPHA FTIR spectrometer (Ettlingen, Germany) in attenuated total reflection (ATR) mode (equipped with a diamond crystal). Processing the spectra was performed using the Grams 9.1 program (Thermo Fisher Scientific, Waltham, MA, USA). The second derivative spectra were obtained using the Savitski–Golay method, with 21-point smoothing. The two-dimensional correlation (2D-COS) intensities were calculated in MATLAB program using the generalized 2D correlation method developed by Noda [67].

Information on the surface elemental composition and chemical states of the elements present at the sample surface was derived from XPS measurements carried out using a PHI 5000 VersaProbe photoelectron spectrometer (Ulvac-PHI, Chansen, MN, USA). The XPS system was equipped with a monochromated Al Kα X-ray source (1486.7 eV). The photoelectron take-off angle was kept at 45° and the vacuum kept at 2 × 10^−6^ Pa during the measurements. The calibration of the binding energy (BE) scale was performed by considering the BE of the C 1s peak (284.6 eV) from the carbon contamination layer. Peak deconvolution was conducted using the PHI MultiPak software, version 9.1.

The dynamic mechanical analysis (DMA) tests were carried out in tension mode on a PerkinElmer Diamond (Waltham, MA, USA) instrument by using polymeric films with a length of 10 mm, a width of 10 mm and a thickness in the range of 0.1–0.2 mm. A temperature scan from −150 °C up to over 350 °C, at a heating rate of 2 °C/min, was applied. The experiment was conducted at 1 Hz. The variation in the storage modulus (E′), loss modulus (E″) and loss tangent (tan δ) as a function of the temperature was obtained. The drops in the E′ curves and the peaks in the E″ and tan δ plots report the relaxations in the polymers. The multi-frequency experiments (0.5, 1, 2, 5, 10 Hz) were effectuated in the same temperature range, with 2 °C/min. The apparent activation energy of the relaxations was determined using the Arrhenius equation:

f = A exp(−E_a_/RT)

using the plot of the frequency logarithm vs. the reciprocal temperature of relaxation:

lnf = lnA − (E_a_/RT)

where f is the frequency (Hz), A is the pre-exponential factor (Hz), R is the gas constant (8.314 J·mol^−1^ K^−1^), T is the absolute temperature (K) and E_a_ is the activation energy (J·mol^−1^). The PICo0 and PICo15 films were subjected to consecutive thermal processes: heating 1 (−150 °C ÷ 125 °C) − isothermal (125 °C, 10 min) − cooling (125 °C ÷ −150 °C) − heating 2 (−150 °C ÷ 350 °C), with 2 °C/min.

The multi-frequency experiments were also performed by increasing the temperature in step-scan mode, with steps of 2 °C, a soak time of 300 s and working frequencies 0.01, 0.02, 0.05, 0.1, 0.2, 0.5, 1, 2, 5, 10 and 20 Hz.

## 3. Results and Discussion

### 3.1. Structural Characterization of the Polyimide Films Using FTIR

**Figure 1:**
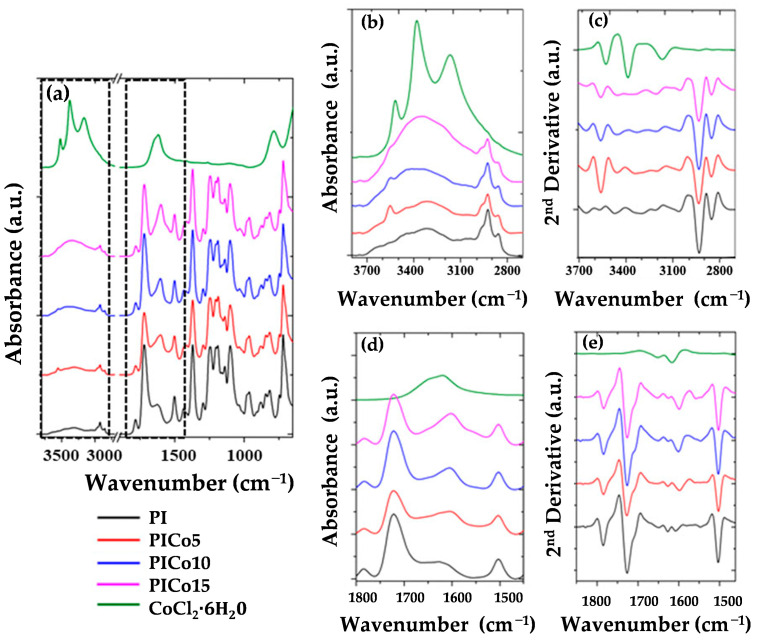
FTIR spectra of PI, CoCl_2_·6H_2_O, PICo5, PICo10 and PICo15 hybrid materials: 3700–700 cm^−1^ spectral region (**a**), 3700–2700 cm^−1^ spectral region (**b**) and its second derivative (**c**) and 1800–1400 cm^−1^ spectral region (**d**) and its second derivative (**e**).

The FTIR spectra of the polyimides are presented in Figure 1a. The regions 3800–2700 cm^−1^ and 1800–1400 cm^−1^ are enlarged in Figure 1b,d. Generally, the second derivative curves (Figure 1c,e) resolve the overlapped component bands. The 3318 cm^−1^ broad characteristic band (Figure 1b) is assigned to the stretching vibration of the OH and NH groups, while the succession of bands identified at 3070, 2958, 2925 and 2855 cm^−1^ is associated with the symmetric and asymmetric vibration of CH from the methyl or methylene groups of the aromatic and aliphatic structures.

In the 1800–1400 cm^−1^ spectral region (Figure 1d), specific imide bands are located at 1785 cm^−1^ (asymmetric stretching vibration of the C=O groups), 1721 cm^−1^ (symmetric stretching vibration of the C=O groups) and 1636 cm^−1^ (bending vibration of the NH groups). Also, the following bands are characteristic of polyimide structures: 1373 cm^−1^ (stretching vibration of the C-N groups) and 720 cm^−1^ (bending vibration of the C=O groups) (Figure 1a). Moreover, the bands located at 1597 cm^−1^ and 1503 cm^−1^ correspond to the quadrant stretching of the carbon-to-carbon double bonds in the aromatic ring and at 1437 cm^−1^ to the stretching vibrations of the C-H groups from the aromatic ring (Figure 1e). The band found at 1247 cm^−1^ corresponds both to the stretching vibrations of the C(CF_3_) groups and the stretching vibrations of the (CO)_2_NC imide groups. The latter are also identified at 1205 cm^−1^ [42,50,68].

The FTIR spectra and second derivative spectra of the polyimides containing varying CoCl_2_ contents (Figure 1b,c) show significant differences in the 3600–2700 cm^−1^ and 1800–1400 cm^−1^ spectral regions. In the first mentioned spectral region, the band assigned to the free and bonded OH and NH groups shifts to a higher wavenumber, from 3320 cm^−1^ to 3355 cm^−1^. This fact indicates that the amic acid groups were involved in the formation of hydrogen bonds. At the same time, a new band located at 3647 cm^−1^ emerges. This can be assigned to the free OH groups that overlap with the bands assigned to the CoCl_2_, which can be observed in all the polyimide–CoCl_2_ systems. In the second mentioned spectral region, a shift to a lower wavenumber (1600 cm^−1^) of the band located at 1636 cm^−1^ is evidenced. The second derivatives of this region (Figure 1e) indicate the presence of five overlapping bands between 1670 and 1530 cm^−1^: 1648, 1628, 1607, 1579 and 1545 cm^−1^. With an increasing CoCl_2_ content, the band located at 1648 cm^−1^ is shifted to 1653 cm^−1^, and the band located at 1607 cm^−1^ is shifted to 1599 cm^−1^. This result indicates that the addition of CoCl_2_ can cause modifications in the structure of the polymeric films.

The frequency shift can be correlated with the level of specific molecular interactions. An alternative view is that the apparent peak position shift is caused by the change in the population of different chemical species. In this case, the position of the peak maximum tends to shift due to the variation in the relative intensity contributions of closely overlapped bands, with their individual frequencies essentially unchanged.

**Figure 2:**
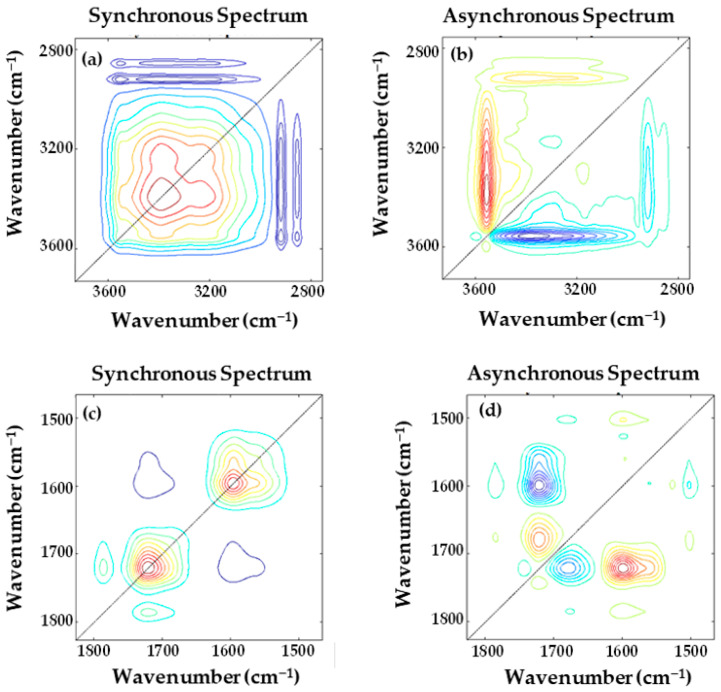
2D-COS spectra for 3700–2750 cm^−1^ region (**a**,**b**) and 1850–1400 cm^−1^ region (**c**,**d**) for PICo15 polymer film.

In order to increase the spectral resolution and to simplify the interpretation of the spectra, generalized two-dimensional IR correlation spectroscopy was applied (Figure 2a–d).

The synchronous spectrum in the 3700–2750 cm^−1^ region shows a large auto-peak at 3384 cm^−1^ and two negative cross-peaks at 3348 vs. 2921 and 2858 cm^−1^. Thus, it seems that this auto-peak consists of more than one peak. The observed negative cross-peak indicates the variation in the opposite direction of the bands involved in its formation. It is known that the asynchronous spectrum has a deconvolution capacity. In this case, the broad band located at 3384 cm^−1^ is split into five bands: 3598, 3556, 3384, 3294 and 3176 cm^−1^. The first and second ones, located at 3598 and 3556 cm^−1^, correspond to the stretching vibrations of free OH and NH groups. The third band, at 3384 cm^−1^, corresponds to the stretching vibrations of H-bonded OH groups, overlapping with the bands assigned to the CoCl_2_. The fourth band (3294 cm^−1^) and the fifth band (3176 cm^−1^) correspond to H-bonded NH groups [59].

Two auto-peaks (1720 cm^−1^ and 1597 cm^−1^) were observed in the synchronous spectrum constructed for PI-CoCl_2_, in the region 1850–1450 cm^−1^: one positive cross-peak at 1785 vs. 1720 cm^−1^ and one negative-cross-peak at 1720 vs. 1597 cm^−1^. The sign of the cross-peaks indicates that the bands located at 1720 and 1785 cm^−1^ vary in the same direction and in the opposite direction to the band located at 1597 cm^−1^. As observed from the asynchronous spectrum, the broad bands centered at 1720 cm^−1^ and 1597 cm^−1^ are each composed of three component bands: at 1743, 1722 and 1678 cm^−1^ and at 1595, 1560 and 1502 cm^−1^, respectively. The bands at 1785 cm^−1^ and 1722 cm^−1^ can be assigned to the asymmetric and symmetric stretching of the C=O groups from the cyclic imide [45,62]. The band located at 1743 cm^−1^ can be assigned to the symmetric stretching vibration of the C=O groups in the intermolecular links and the band at 1678 cm^−1^ to the stretching vibration of the C=O groups from the amide I linkage (amic acid groups). The band located at 1597 cm^−1^ corresponds to the quadrant stretching of the carbon-to-carbon double bonds in the aromatic ring, which overlaps in part with the vibration bands of amide I [45] and CoCl_2_. The band found at 1502 cm^−1^ is assigned to the ring-breathing modes of the para-substituted benzene ring [39], while the one found at 1560 cm^−1^ can be assigned to the stretching of NH in the amide that belongs to the amic acid groups [69]. Therefore, 2D IR correlation spectroscopy suggests that amic acid groups and intermolecular links are present together with the imide ring in the PI-CoCl_2_ samples.

Analysis of the synchronous and asynchronous 2D correlation spectra revealed that the bands associated with the imide C=O groups are changed before the bands corresponding to the C=O groups from the amide and the intermolecular links and also before the bands located at 1600 cm^−1^, which are assigned to the stretching vibrations of the C=C groups and amide I. This fact indicates that the formation of the imide ring occurs before the changes in the amide groups.

**Figure 3:**
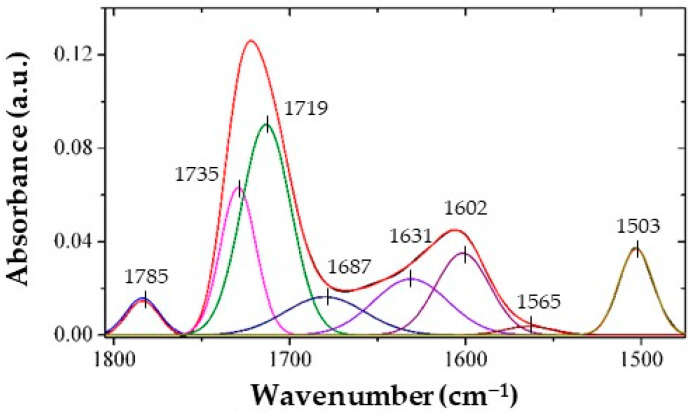
Deconvoluted IR spectra in the 1800–1400 cm^−1^ region for PICo15 polymer film. The main IR curve is red and the others are obtained after the deconvolution process.

The IR spectra of the PICo15 sample were deconvoluted in the 1800–1400 cm^−1^ region according to the observed band in the asynchronous spectrum, using the method proposed by Niu et al. [70] (Figure 3).

Ten bands were obtained after the deconvolution process: 1785, 1735, 1719, 1687, 1657, 1631, 1602, 1565, 1531 and 1503 cm^−1^. The bands’ positions remained constant for all the studied samples and differences appear only in the band area and/or band width. The most important information for our rationale was included in Table 1.

When the areas of the bands associated with the asymmetric and symmetric vibrations of the C=O groups (1784 cm^−1^ and 1719 cm^−1^) are compared with the area assigned to the amic acid groups (1630 cm^−1^), it becomes evident that the polyimide contains a non-negligible quantity of unreacted amic acid. This represents a hint that the imidization was incomplete. Moreover, both the aromatic C=C groups and the CoCl_2_ are responsible for the 1604 cm^−1^ band. However, intuitively, the increase in the area of this band with the quantity of CoCl_2_ is credited mainly to the salt, which interacts in some way with the polymer.

### 3.2. X-ray Photoelectron Spectroscopy

The nature of the cobalt and the coordination geometry of the potential donor atoms from the polyimide films (N, O) to the cobalt were studied using XPS. Differences in the coordination and oxidation states are expected to exist in the chemical shifts, satellite structure and valence band structure.

**Figure 4:**
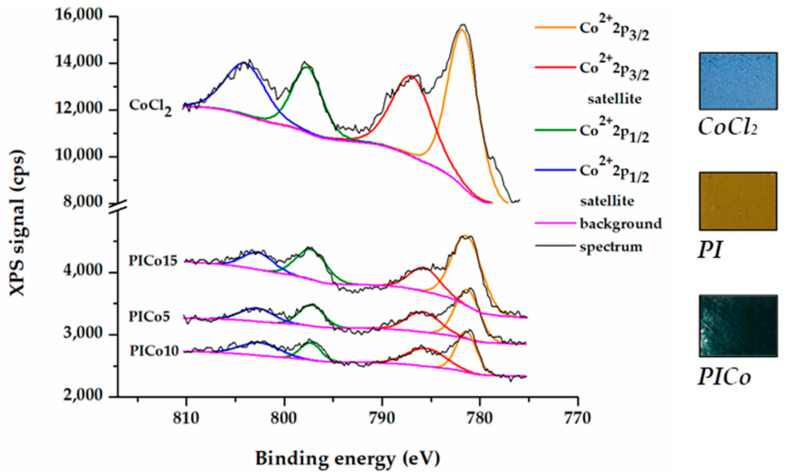
Deconvoluted 2p X-ray photoelectron spectra of CoCl_2_ and PICo5, PICo10 and PICo15 films. Images of the anhydrous CoCl_2_ and the polyimide films without (PI) and with additives (PICo) are on the right side of the figure.

The Co 2p XPS spectrum exhibits two major peaks, with the binding energy values at 797.5 eV and 781.5 eV attributed to the Co 2p_1/2_ and Co 2p_3/2_ spin-orbit peaks, respectively (Figure 4).

The Co 2p_1/2_−Co 2p_3/2_ energy separations are around 16 eV, and two strong associated satellites are located at the high-binding-energy side of the main peaks (Table 2).

All of these features are commonly found for high-spin Co (II) compounds [15,71,72]. After the polyimides were doped with CoCl_2_, the main 2p peaks have broadened and slightly changed. The presence of strong satellite bands indicates that the oxidation state of the cobalt was not changed: it is equal to +2. It is known that cobalt compounds with a +3 oxidation state have very weak satellite peaks or do not have them at all [11].

High-spin cobalt compounds usually present a tetrahedral or octahedral geometry. The blue color of the anhydrous CoCl_2_ is ascribed to the formation of tetrahedral complexes. The appearance, position and binding energy values, almost consistent with those in CoCl_2_, indicate a predominant tetrahedral environment around the cobalt in the Co-doped PI. This feature is also confirmed by the green color of the PI-CoCl_2_ films (the blue color of CoCl_2_ combined with yellow color of the polyimide film). The presence of the tetrahedral environment around Co^2+^ was also confirmed using UV–Vis absorption spectroscopy [66]. The absorbance from the tetrahedral structure is more intensive than that of the octahedral structure, which almost disappeared.

For a complete characterization of the electronic and chemical state of the cobalt, analysis of the N 1s and O 1s peaks is necessary. The N spectrum is composed of a single component at 400 eV corresponding to the nitrogen of the imide groups. The O spectrum is composed of two components at 531.8 eV and 533.5 eV, assigned to the carbonyl oxygens from the imide rings and the oxygen coming from the ether, respectively (Table 2).

A positive binding energy shift for nitrogen and negative shift for oxygen are observed for the cobalt-doped PI relative to the undoped PI. The positive shift for nitrogen is consistent with the decrease in the electronic density upon the donation of its lone pair of electrons to the cobalt [73]. So, the XPS results indicate that Co(II)–N coordinated bonds were formed. The coordination of an oxygen atom to a metal ion results in an increase in binding energy [35]. In our case, a negative shift for oxygen can be rationalized as the capacity of the cobalt to coordinate with the amic acid groups.

### 3.3. Dynamic Mechanical Analysis

Dynamic mechanical analysis provides information on the relaxation behavior of polymers, which depends on the local molecular environment. Therefore, the DMA results can offer an understanding of the coordination linkage and reinforcement mechanism of the cobalt ions in a polyimide, if they exist. Before discussing the doped polymers, detailed comments on the viscoelastic behavior of undoped polyimide are necessary.

**Figure 5:**
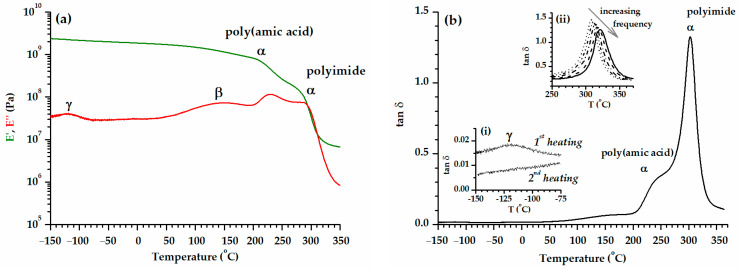
The variation in E′, E″ (**a**) and tan δ (**b**) with temperature, at 1 Hz and 2 °C/min, for the sample PI. Details of the γ-relaxation region is represented in (**b**), inset (i). The plot of tan δ in the glass transition region, obtained at five frequencies (0.5, 1, 2, 5, 10 Hz), is displayed in (**b**), inset (ii).

The DMA behavior of PI is represented in Figure 5, and the main viscoelastic characteristics are included in Table 3.

A broad γ-relaxation is noticed in the sub-glass transition region, centered around –113 °C (tan δ peak), and is associated with the presence of residual water in the film [74,75,76]. This peak disappears in the second heating run of the heating–cooling–heating experiment (Figure 5(bi)). Further on, a large β-relaxation begins around 50 °C and spans a range close to 200 degrees. Generally, the β-relaxation of polyimides is attributed to the rotational vibrations of various groups [77,78]. During sub-glass transition relaxations, the storage modulus E′ decreases by less than a factor of two.

An important drop in the storage modulus starts around 210 °C and represents the onset of the α-relaxation. During the α-relaxation, long-range coordinated molecular motions take place. The decrease in the E′ modulus in two steps is the result of two different molecular motions. Two peaks are also noticed on the E″ and tan δ curves. This means that the imidization was not complete, and the final polyimide includes also amic acid segments, which usually relax at a lower temperature than imide polymer segments (Table 3). It is not excluded that imidization continues even in the instrument oven.

The characteristics of the viscoelastic parameters E′, E″ and tan δ during the glass transition region depend greatly on the polymer structure. The maximum value of the tan δ peak is higher than 1 (h_tanδ_, Table 3). This is also reflected in the fact that E′ and E″ intersect two times during the α-relaxation (Figure 5a). Therefore, there are no constraints on the structure of the polyimide that would determine E′ > E″, i.e., a tan δ peak (h_tanδ_) value smaller than 1. Also, the tan δ peak decreases with an increasing frequency due to the fact that no secondary processes overlap with the α-relaxation, which would confer additional mobility to the polymer chain [79]. The E′ modulus of the rubbery plateau remains constant until the end of the experiment.

**Figure 6:**
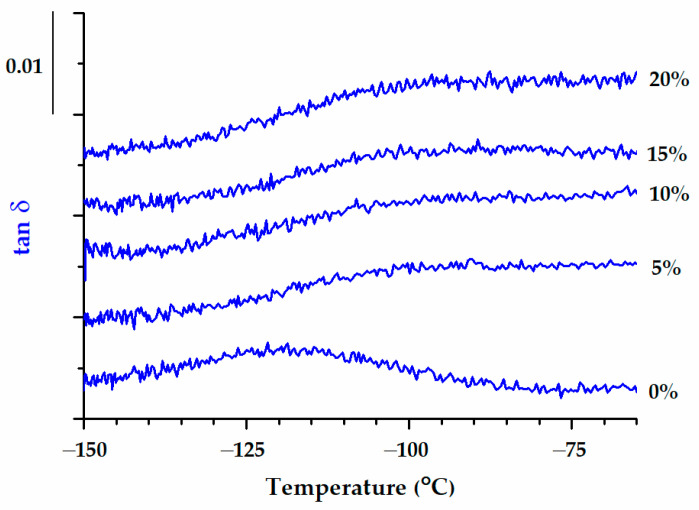
The γ-relaxation region for the polyimides with different contents of CoCl_2_. For clarity, the tan δ curves were vertically shifted.

**Figure 7:**
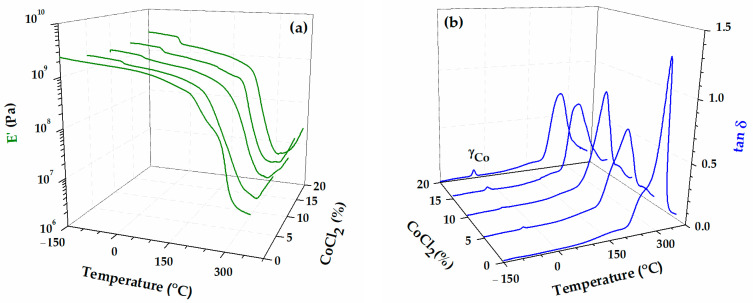
The variation in the E′ modulus (**a**) and tan δ (**b**) for the polyimides with different contents of CoCl_2_. For comparison, polyimide with no CoCl_2_ was also included.

The viscoelastic behavior of the cobalt-doped MMDA-6FDA polyimide is presented below. At least theoretically, the cobalt can coordinate with both nitrogen and oxygen atoms, which belong to one or two polymer chains. In the sub-glass transition region, when compared with the viscoelastic behavior of PI, a shift in the γ-relaxation to a higher temperature (Figure 6) and the presence of a new relaxation around −40 °C (γ_Co_, Figure 7a,b) are noticed. Figure 7a,b do not include the E″ modulus curves in order to keep the figures clear.

The γ-relaxation shift associated with residual water does not depend on the cobalt content. Bas et al. associated this shift with an increase in chain packing [76]. The new γ_Co_ relaxation can be attributed only to the inclusion of cobalt in the structure of the polymer. As far as we are aware, this relaxation has not yet been reported in the literature. Certain features distinguish this relaxation. The E′ modulus for PICo20 decreases 1.5 times during this relaxation (Table 3). Actually, PICo20 is the only sample that registers a higher E′ modulus than PI, but only at very low temperature values. After this relaxation, all the samples have an E′ modulus lower than that of the PI with no cobalt (Table 3). It is important to mention the large value of the activation energy for the γ_Co_ relaxation, which is a secondary relaxation (Figure S1, Supplementary Information). The γ_Co_ relaxation is present in the second heating stage, but is shifted to higher temperature (Figure S2, Supplementary Information), most probably as a result of the elimination of water.

The onset temperature of the glass transition (E′ onset) decreased initially when a small quantity of CoCl_2_ was included in the polyimide, but it had an increasing trend with the content of the cobalt. The glass transition region comprises two steps for all the samples, meaning that not all the amic acid groups were converted into imide groups. An accurate assessment of the glass transition temperature cannot be realized during a thermal scan experiment if additional imidization cannot be excluded [47,80,81]. Due to the additional imidization during the DMA experiment, the glass transition temperature of the incompletely imidized PI increases continually. The values of the glass transition temperatures estimated from the E″ and tan δ peaks are not absolute but represent the glass transition temperature values at a certain moment. Since all the experiments were performed in the same conditions, the T_g_ values of the amic acid and imide segments determined from the tan δ peaks are included in Table 3 for comparison. In this situation, if we want to talk about the glass transition temperature of the initial materials, the partially imidized polyimides as they resulted from synthesis, the temperatures of E′ onset are the most suitable.

The tan δ peak decreases as more dopant is included in the film. The presence of cobalt limits the cooperative segmental motions of the polymer chains; a higher cobalt content makes this effect stronger. At some point, the tan δ peaks of the amic acid and imide segments merge, and they cannot be separated anymore. The polyimide becomes a heterogeneous structure with overlapping glass transitions.

**Figure 8:**
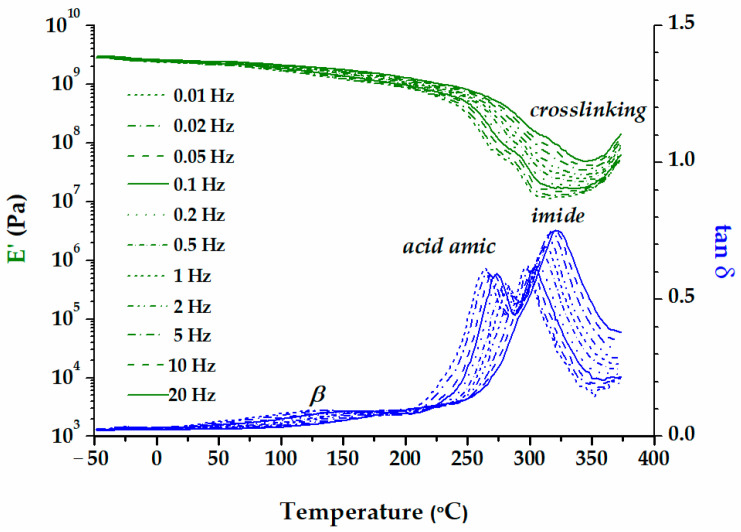
The variation in E′ and tan δ for the sample PICo15 obtained in a multi-frequency experiment. The explanation of the curves is the same for the green lines (E’) and for the blue lines (tan δ).

Typically, low heating rates and low frequencies separate better overlapping processes [82,83,84]. A step scan experiment performed for the sample PICo15 resolves the large tan δ peak that emerges in the isochronal ramp experiment in two peaks (Figure 8).

**Figure 9:**
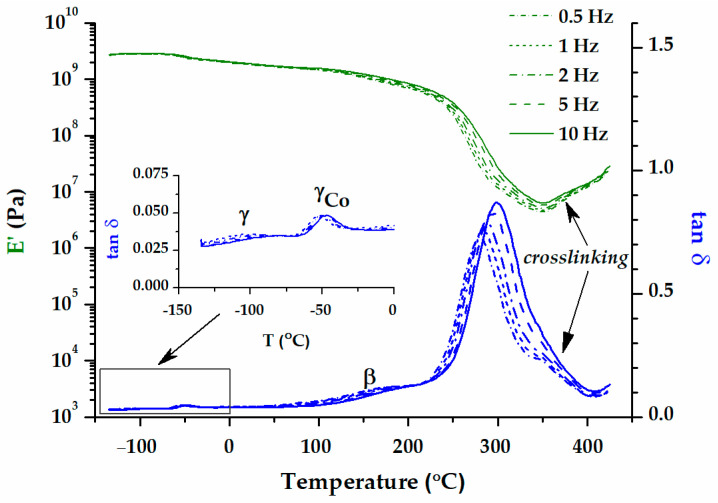
The variation in E′ (green lines) and tan δ (blue lines) for the sample PICo10 obtained in an experiment performed at five frequencies.

The first and second peaks are associated with the relaxation of the amic acid and imide chain segments, respectively. The rise in the E′ modulus after the glass transition is an indication of an increase in the film’s rigidity. It seems that metal ions form crosslinks with polyimide chains over 320 °C. The same has already been reported for CoCl_2_-modified N-picolyl polyurethane and NBR [32,33]. However, this gain in rigidity more likely starts in the last part of the glass transition process. Initially, the descent in E′ is leveled out, and then it takes an upturn trend. In the experiment performed at five frequencies, this process is reflected as a shoulder on the descending side of the tan δ peak, as is represented in Figure 9 for PICo10.

This shoulder does not have the characteristics of a relaxation (frequency-independent) [53,82] and occurs in the same temperature range as the E′ increase. The increase in rigidity is more intense and starts at a lower temperature, as the content in CoCl_2_ is higher.

The last point in question is why the storage modulus and the glass transition temperature are lower for the doped polymers as compared to the undoped polymers. (Table 3). Amic acid–imide structures are known for their hydrogen bond intermolecular interactions and the π-π interactions between the benzene rings of diphenylmethane. Coordination with the cobalt can break the physical interactions between the macromolecular chains and may result in a decrease in both the storage modulus and glass transition temperature of the doped polyimides.

## 4. Conclusions

Using several combined experimental techniques (DMA, the second derivative FTIR and 2D-COS), we analyzed the structure and mechanical behavior of the CoCl_2_–polyimide systems. The experiments demonstrated that in the systems we studied, the thermal imidization was incomplete. The spectral vibrational configurations are modified as the quantity of CoCl_2_ is increased in a way that does not preclude an interaction between the salt and the polyimides. With the help of XPS, it was possible to identify a tetrahedral environment around the cobalt in the polyimide films and Co(II)–N coordinated bonds. The coordination of the cobalt with the residual amic acid groups is possible. A specific relaxation (γ_Co_) was revealed using DMA in the CoCl_2_–polyimide films, around −40 °C. However, the effect of CoCl_2_ on the film stiffness was not straightforward due to two competing mechanisms of its interaction with the polymer network. In the first instance, at a low CoCl_2_ content, the salt triggers disruption of the intermolecular hydrogen bonds and/or π-π interactions. This is reflected both in the smaller values of the E′ modulus and glass transition temperature as compared with those of pristine polyimide. The stiffness of the films increased when the content of CoCl_2_ went over 15%. Also, raising the temperature over 320 °C in the DMA oven entailed in the presence of CoCl_2_ the formation of a crosslinked structure.

## Figures and Tables

**Table 1:** The area of the main bands that are assigned to polyimide structure.

Sample	Band Area				
	1784 cm^−1^	1719 cm^−1^	1630 cm^−1^	1604 cm^−1^	1555 cm^−1^
PI	4.7	41.2	12.8	5.9	-
PICo5	3.8	32.7	10.6	17.9	0.3
PICo10	5.4	42.9	8.3	16.8	2.0
PICo15	4.7	35.3	8.4	25.3	4.1

**Table 2:** Binding energies (eV) of the component elements of the undoped and doped films *.

Sample	N 1s	O 1s		Co 2p			
		Ether	Imide	Satellite 1	Co 2p_1/2_	Satellite 2	Co 2p_3/2_
CoCl_2_	-	-	-	803.5	797.5	787.0	781.5
PI	399.7	533.5	531.8	-	-	-	-
PICo5	400.0	533.4	531.8	802.9	797.3	785.8	781.3
PICo10	399.8	533.4	531.8	802.6	797.3	785.3	781.5
PICo15	399.7	533.2	531.6	802.8	797.4	785.5	781.5

* ±0.2 eV.

**Table 3:** The main viscoelastic characteristics of the polyimides as derived from the DMA experiment.

Sample	E′ (GPa) Glassy Region		α-Relaxation Onset		tan δ α-Relaxations			E′ (MPa) Rubbery Plateau	R ^6^
	–140 °C ^1^	20 °C ^2^	E′ (GPa)	T (°C)	T (°C) ^3^	T (°C) ^4^	h_tanδ_^5^		
PI	2.33	1.80	0.72	210	238	305	1.30	6.7 (350 °C)	108.0
PICo5	1.88	1.25	0.50	195	225	279	0.69	5.2 (340 °C)	96.0
PICo10	1.80	1.10	0.42	205	-	285	0.89	6.3 (340 °C)	66.5
PICo15	1.95	1.14	0.46	202	-	266	0.70	6.6 (325 °C)	70.0
PICo20	2.76	1.37	0.40	217	-	272	0.70	5.0 (310 °C)	80

^1^ The temperatures correspond to the region before γ-relaxation; ^2^ the temperatures correspond to the region before β-relaxation; ^3^ the tan δ peak associated with amic acid chain segments; ^4^ the tan δ peak associated with imide chain segments; ^5^ the height of tan δ peak associated with imide chain segments; ^6^ the ratio between the E′ moduli before α-relaxation and E′ of the rubbery plateau.

## Data Availability

The data presented in this study are available on request from the corresponding author.

## Supplementary Materials

The following supporting information can be downloaded at https://www.mdpi.com/article/10.3390/ma17030753/s1. Figure S1: The Arrhenius plot of the γ_Co_ secondary relaxation for the samples PICo5, PICo10, PICo15 and PICo20 and the corresponding activation energy (Ea); Figure S2: The γ_Co_ relaxation for the sample PICo15 during the first heating stage and the second heating stage. The isochronal experiment (1 Hz) was performed with 2 °C/min during all heating and cooling stages. The first heating stage was stopped at 125 °C, and the sample was maintained 10 min at this temperature before the cooling.

## References

[1] Sroog C.E., Ghosh M.K., Mittal K.L. (1996). History in the invention and development of the polyimides. Polyimides. Fundamentals and Applications.

[2] Liaw D.J., Wang K.L., Huang Y.-C., Lee K.-R., Lai J.-Y., Ha C.-S. (2012). Advanced polyimide materials: Synthesis, physical properties and applications. Prog. Polym. Sci..

[3] Damaceanu M.-D. (2022). Progress on polymers containing imide rings for advanced technologies: A contribution from ICMPP of the Romania Academy. Chemistry.

[4] Jiang L.Y., Wang Y., Chung T.-S., Qiao X.Y., Lai J.-Y. (2009). Polyimides membranes for pervaporation and biofuels separation. Prog. Polym. Sci..

[5] Verbiest T., Burland D.M., Jurich M.C., Lee V.Y., Miller R.D., Volksen W. (1995). Exceptionally thermally stable polyimides for second-order nonlinear optical applications. Science.

[6] Goh P.S., Ismail A.F., Sanip S.M., Ng B.C., Aziz M. (2011). Recent advances of inorganic fillers in mixed matrix membrane for gas separation. Sep. Purif. Technol..

[7] Li J., Qi S., Li J., Zhang M., Wang Z. (2015). A highly thermostable and transparent lateral heat spreader based on silver nanowire/polyimide composite. RSC Adv..

[8] Rancourt J.D., Taylor L.T. (1987). Preparation and properties of surface-conductive polyimide films via in situ codeposition of metal salts. Macromolecules.

[9] Ding D., Yan X., Zhang X., He Q., Qiu B., Jiang D., Wei H., Guo J., Umar A., Sun L. (2015). Preparation and enhanced properties of Fe_3_O_4_ nanoparticles reinforced polyimide nanocomposites. Superlattices Microstruct..

[10] Kaburagi Y., Hataori H., Yoshida A., Hishiyama Y., Inagaki M. (2001). Carbon films containing transition metal particles of nano and submicron sizes. Synth. Met..

[11] Khor E., Taylor L.T., Sheats J.E., Carraher C.E., Pittman C.E. (1985). A study of cobalt containing polyimide films. Metal-Containing Polymeric Systems.

[12] Sawada T., Ando S. (1998). Synthesis, characterization and optical properties of metal-containing fluorinated polyimide films. Chem. Mater..

[13] Mu S., Wu Z., Wang Y., Qi S., Yang X., Wu D. (2010). Formation and characterization of cobalt oxide layers on polyimide films via surface modification and ion-exchange technique. Thin Solid Films.

[14] Maya E.M., de la Torre G., Lozano A.E., Torres T., de la Campa J.G., de Abajo J. (2006). Novel cobalt (II) phthalocyanine-containing polyimides: Synthesis, characterization, thermal and optical properties. Macromol. Rapid Commun..

[15] Rancourt J.D., Taylor L.T., Dickie R.A., Labana S.S., Bauer R.S. (1988). Characterization of cobalt (II) chloride-modified condensation polyimide films—Properties before and after solvent extraction. Cross-Linked Polymers: Chemistry, Properties and Applications, ACS Symposium Series.

[16] Bushkova O.V., Koryakova I.P., Skorik Y.A., Lirova B.I., Pestov A.V., Zhukovsky V.M. (2008). Influence of metal coordination on conductivity behavior in poly(butadiene-acrylonitrile)-CoCl_2_ system. Electrochim. Acta.

[17] Gao R., Wang K., Li Y., Wang F., Sun W.-H., Redshaw C., Bochmann M. (2009). 2-Benzoxazolyl-6-(1-(arylimino)ethyl)pyridyl cobalt (II) chlorides: A temperature switch catalyst in oligomerization and polymerization of ethylene. J. Mol. Catal. A-Chem..

[18] Sister V.G., Kosivtsov Y.Y., Sul’man E.M., Lugovoi Y.V., Ivannikova E.M., Yamchuk A.I. (2011). Effect of cobalt chloride on the physical and chemical properties of the solid residue from the pyrolysis of polymer tire cord. Theor. Found. Chem. Eng..

[19] Li F., Arthur E.E., La D., Li Q., Kim H. (2014). Immobilization of CoCl_2_ (cobalt chloride) on PAN (polyacrylonitrile) composite nanofiber mesh filled with carbon nanotubes for hydrogen production from hydrolysis of NaBH_4_ (sodium borohydride). Energy.

[20] Naboka O., Sanz-Velasco A., Lundgren P., Enoksson P., Gatenholm P. (2012). Cobalt (II) chloride promoted formation of honeycomb patterned cellulose acetate films. J. Colloid Interf. Sci..

[21] Young J.A. (2003). Cobalt(II) Chloride Hexahydrate. J. Chem. Educ..

[22] Mishra S.K., Kanungo S.B. (1992). Thermal dehydration and decomposition of cobalt chloride hydrate (CoCl_2_·xH_2_O). J. Therm. Anal..

[23] Tay C.M., Tan K.M., Tjin S.C., Chan C.C., Rahardjo H. (2004). Humidity sensing using plastic optical fibers. Microw. Opt. Technol. Lett..

[24] Zidan H.M., Abu-Elnader M. (2005). Structural and optical properties of pure PMMA and metal chloride-doped PMMA films. Physica B.

[25] Ahmed R.M. (2014). Optical properties and structure of cobalt chloride doped PVA and its blend with PVP. Int. J. Mod. Phys. B.

[26] Abiona A.A., Ajao J.A., Chigome S., Kana J.B.K., Osinkolu G.A., Maaza M. (2010). Synthesis and characterization of cobalt chloride/poly(ethylene oxide) electrospun hybrid nanofibers. J. Sol-Gel Technol..

[27] Shao C., Wang Q., Mao Y., Li Q., Wu C. (2019). Influence of carbon nanotubes content on the properties of acrylonitrile-butadiene rubber/cobalt chloride composites. Mater. Res. Express.

[28] Zia M.A., Khosa M.K., Noor A., Qayyum S., Shakir M.S. (2022). PMMA/ABS/CoCl_2_ composites for pharmaceutical applications: Thermal, antimicrobial, antibiofilm, and antioxidant studies. Molecules.

[29] Ahmed R.M., Abou-Laila M.T., Taha E.O. (2023). Investigating into physical properties of composites of polymer blends and cobalt chloride irradiated by gamma ray for optical devices development. Mater. Today Commun..

[30] Belfiore L.A., McCurdie M.P., Ueda E. (1993). Polymeric coordination complexes based on cobalt, nickel, and ruthenium that exhibit synergistic thermal properties. Macromolecules.

[31] Guan H.-M., Cheng X.-S. (2004). Study of cobalt(II)-chitosan coordination polymer and its catalytic activity and selectivity for vinyl monomer polymerization. Polym. Adv. Technol..

[32] Shen Q.-D., Yang C.-Z. (1998). Transition metal complexes of N-picolyl polyurethane. J. Polym. Sci. B Polym. Phys..

[33] Huang J., Qian S., Shen F., Wu C. (2007). Investigation on the coordination interfacial reaction by dynamical mechanical analysis. J. Macromol. Sci. B.

[34] Sava I., Chisca S., Bruma M., Lisa G. (2010). Comparative study of aromatic polyimides containing methylene units. Polym. Bull..

[35] Bogges R.K., Taylor L.T. (1987). Characterization of cobalt-modified polyimides. J. Polym. Sci. A Polym. Chem..

[36] Varma I.K., Saxena S., Tripathi A., Goel T.C., Varma D.S. (1986). Effect of metal halides on thermal, mechanical, and electrical properties of polypyromellitimide films. J. Appl. Polym. Sci..

[37] Halper S.R., Villahermosa R.M. (2009). Cobalt-containing polyimides for moisture sensing and absorption. ACS Appl. Mater. Inter..

[38] Thomson B., Bartges D., Czerniawski D., Painter P.C. (1989). FTIR studies of polyimides: Thermal curing. Macromolecules.

[39] Diaham S., Locatelli M.L., Lebey T., Malec D. (2011). Thermal imidization optimization of polyimide thin films using Fourier transform infrared spectroscopy and electrical measurements. Thin Solid Films.

[40] Kumar A., Tateyama S., Yasaki K., Ali M.A., Takaya N., Singh R., Kaneko T. (2016). ^1^H NMR and FT-IR dataset based structural investigation of poly(amic acid)s and polyimides from 4,4′-diaminostilbene. Data Brief.

[41] Shin T.J., Ree M. (2007). Thermal imidization and structural evolution of thin films of poly(4,4′-oxyduphenylene p-pyromellitamic diethyl ester). J. Phys. Chem. B.

[42] Thomson B., Park Y., Painter P.C., Snyder R.W. (1989). Hydrogen bonding in poly(amic acid)s. Macromolecules.

[43] Guerra G., Choe S., Williams D.J., Karasz F.E., MacKnight W.J. (1988). Fourier transform infrared spectroscopy of some miscible polybenzimidazole/polyimide blends. Macromolecules.

[44] Shin T.J., Ree M. (2002). Poly(3,4′-oxydiphenylene pyromellitamic acid) 1. Time-resolved infrared spectroscopic study of thermal imidization in thin films. Macromol. Chem. Phys..

[45] Shin T.J., Lee B., Youn H.S., Lee K.-B., Ree M. (2001). Time-resolved synchrotron X-ray diffraction and infrared spectroscopic studies of imidization and structural evolution in a microscaled film of PMDA-3,4′-ODA poly(amic acid). Langmuir.

[46] Lockwood D.J. (1973). Lattice vibrations of CdCl_2_, CdBr_2_, MnCl_2_, and CoCl_2_: Infrared and Raman spectra. J. Opt. Soc. Am..

[47] Cho D., Choi Y., Drzal L.T. (2001). Simultaneous monitoring of the imidization and cure of LaRC PETI-5 sized on a braided glass fabric substrate by dynamic mechanical analysis. Polymer.

[48] Cristea M., Ionita D., Garbea M., Damaceanu M.-D., Barzic A.I., Rawat N.K., Haghi A.K. (2021). Dynamic molecular phenomena in polyimides investigated by dynamic mechanical analysis. Imidic Polymers and Green Polymer Chemistry.

[49] Chen W.J., Chen W., Zhang B., Yang S., Liu C.-Y. (2017). Thermal imidization process of polyimide film: Interplay between solvent evaporation and imidization. Polymer.

[50] Sava I., Chisca S., Bruma M., Lisa G. (2011). Effect of thermal curing on the properties of thin films based on benzophenonetetracarboxylic dianhydride and 4,4′-diamino-3,3′-dimethyldiphenylmethane. J. Therm. Anal. Calorim..

[51] Lee C.Y.-C., Goldfarb I.J. (1981). The glass transition temperature of partially cured polymers as a non-equilibrium parameter and its effect on observed dynamic mechanical behavior. Polym. Eng. Sci..

[52] Feger C. (1989). Curing of polyimides. Polym. Eng. Sci..

[53] Tyan H.-L., Liu Y.-C., Wei K.-H. (1999). Enhancement of imidization of poly(amic acid) through forming poly(amic acid)/organoclay nanocomposites. Polymer.

[54] Tang Q.-Y., Chen J., Chan Y.C., Chung C.Y. (2010). Effect of carbon nanotubes and their dispersion on thermal curing of polyimide precursors. Polym. Degrad. Stab..

[55] Ikeda S., Akamatsu K., Nawafune H., Nishino T., Deki S. (2004). Formation and growth of copper nanoparticles from ion-doped precursor polyimide layers. J. Phys. Chem. B.

[56] Noda I. (2016). 2DCOS and I. Three decades of two-dimensional correlation spectroscopy. J. Mol. Struct..

[57] Noda I. (2007). Two-dimensional correlation analysis useful for spectroscopy, chromatography, and other analytical measurements. Anal. Sci..

[58] Lai H., Wu P. (2010). A infrared spectroscopic study on the mechanism of temperature-induced phase transition of concentrated aqueous solutions of poly(N-isopropylacrylamide) and N-isopropylpropionamide. Polymer.

[59] Popescu M.-C., Filip D., Vasile C., Cruz C., Rueff J.M., Marcos M., Serrano J.L., Singurel G. (2006). Characterization by Fourier Transform Infrared Spectroscopy (FT-IR) and 2D IR Correlation Spectroscopy of PAMAM dendrimer. J. Phys. Chem. B.

[60] Yu K.H., Yoo Y.H., Rhee J.M., Lee M.-H., Yu S.-C. (2003). Two-dimensional Raman correlation spectroscopy study of the pathway for the thermal imidization of poly(amic acid). Bull. Korean Soc..

[61] Park Y., Noda I., Jung Y.M. (2016). Novel developments and applications of two-dimensional correlation spectroscopy. J. Mol. Struct..

[62] Seo H., Chae B., Im J.H., Jung Y.M., Lee S.W. (2014). Imidization induced structural changes of 6FDA-ODA poly(amic acid) by two-dimensional (2D) infrared correlation spectroscopy. J. Mol. Struct..

[63] Nishikawa Y., Nakano T., Noda I. (2014). Molecular interaction of polyimide films probed by using soft-pulse dynamic compression ATR time-resolved infrared and double Fourier-transform based 2D-IR spectroscopy. Vib. Spectrosc..

[64] Lee Y.J., Chae B., Park Y., Jung Y.M., Lee S.W. (2020). 2D infrared correlation study of the effect of base catalyst on thermal imidization of polyamic acid. J. Mol. Struct..

[65] Damaceanu M.-D., Sava I., Constantin C.-P. (2016). The chromic and electrochemical response of CoCl_2_—Filled polyimide materials for sensing applications. Sens. Actuat. B-Chem..

[66] Sava I., Damaceanu M.-D., Lisa G. (2016). Insights into the physico-chemical behavior of CoCl_2_/polyimide hybrid materials. J. Polym. Res..

[67] Noda I. (1993). Generalized two-dimensional correlation method applicable to Infrared, Raman, and other types of spectroscopy. Appl. Spectrosc..

[68] Zhuang Y., Gu Y. (2013). Poly(benzoxazole-amide-imide) copolymers for interlevel dielectrics: Interchain hydrogen bonding, molecular arrangement and properties. J. Polym. Res..

[69] Sava I., Iosip M.D., Bruma M., Hamciuc C., Robison J., Okrasa L., Pakula T. (2003). Aromatic polyamides with pendent acetoxybenzamide groups and thin films made therefrom. Eur. Polym. J..

[70] Niu H., Huang M., Qi S., Han E., Tian G., Wang X., Wu D. (2013). High-performance copolyimide fibers containing quinazolinone moiety: Preparation, structure and properties. Polymer.

[71] Frost D.C., McDowell I.S. (1972). π for multiple splitting of 2p photoelectron lines of transition metal complexes. Chem. Phys. Lett..

[72] Brown D.G., Weser U. (1979). XPS spectra of spin-triplet cobalt(III) complexes. Z. Naturforsch. B.

[73] Burness J.H., Dillard J.G., Taylor L.T. (1975). An X-ray photoelectron spectroscopic study of cobalt(II) Schiff base complexes and their oxygenation products. J. Am. Chem. Soc..

[74] Rebenfeld L., Makarewicz P.J., Weigmann H.-D., Wilkes G.L. (1976). Interactions between solvents and polymers in the solid state. J. Macromol. Sci. Polym. Rev..

[75] Bernier G.A., Kline D.E. (1968). Dynamic mechanical behavior of a polyimide. J. Appl. Polym. Sci..

[76] Bas C., Tamagna C., Pascal T., Alberola N.D. (2003). On the dynamic mechanical behavior of polyimides based on aromatic and alicyclic dianhydrides. Polym. Eng. Sci..

[77] Tang H., Dong L., Zhang J., Ding M., Feng Z. (1996). Study on the β relaxation mechanism of thermosetting polyimide/thermoplastic polyimide blends. Eur. Polym. J..

[78] Li F., Fang S., Ge J.J., Honigfort P.S., Chen J.-C., Harris F.W., Cheng S.Z.D. (1999). Diamine architecture effects on glass transitions, relaxation processes and other material properties in organo-soluble aromatic polyimide films. Polymer.

[79] Cristea M., Ionita D., Hulubei C., Timpu D., Popovici D. (2011). Chain packing versus chain mobility in semialiphatic BTDA-based copolyimides. Polymer.

[80] Kim K., Ryou J.H., Kim Y., Ree M., Chang T. (1995). Thermal imidization behavior of aromatic poly(amic dialkyl ester) precursors derived from biphenyltetracarboxylic dianhydride. Polym. Bull..

[81] Lee C.Y.C. (1988). Torsion impregnated cloth analysis for resin rheological studies. Polym. Eng. Sci..

[82] Cristea M., Ionita D., Simionescu B.C. (2010). A new insight in the dynamo-mechanical behavior of poly(ethylene terephtalate). Eur. Polym. J..

[83] Stark W. (2013). Investigation of the curing behavior of carbon fibre epoxy prepreg by dynamic mechanical analysis DMA. Polym. Test..

[84] Cristea M., Ionita D., Iftime M.M. (2020). Dynamic mechanical analysis investigations of PLA-based renewable materials: How are they useful?. Materials.

