# Supplementary material for: Temperature-Controlled Chain Dynamics in Polyimide Doped with CoCl2 Probed Using Dynamic Mechanical Analysis
Source: Materials (Basel). 2024 Feb 4;17(3):753. doi: 10.3390/ma17030753 (PMC10856759; doi:10.3390/ma17030753)
Supplement: Supplementary file 1 [file materials-17-00753-s001.zip › materials-2757289-supplementary.pdf]

Supporting Information

Temperature-controlled chain dynamics in polyimide doped with $CoCl_2$ probed using dynamic mechanical analysis

Daniela Ionita [1], Mariana Cristea [1,*], Ion Sava [1], Maria-Cristina Popescu [1], Marius Dobromir [2] and Bogdan C. Simionescu [1]

[1] "Petru Poni" Institute of Macromolecular Chemistry, Aleea Grigore Ghica Voda 41A, 700487 Iasi, Romania; ionita.daniela@icmpp.ro (D.I.); isava@icmpp.ro (I.S.); cpopescu@icmpp.ro (M.-C.P.); bcsimion@icmpp.ro (B.C.S.)

[2] Department of Exact and Natural Sciences, Institute of Interdisciplinary Research, "Alexandru Ioan Cuza" University of Iasi, Blvd. Carol I 11, 700506 Iasi, Romania; marius.dobromir@uaic.ro (M.D.)

* Correspondence: mcristea@icmpp.ro (M.C.)

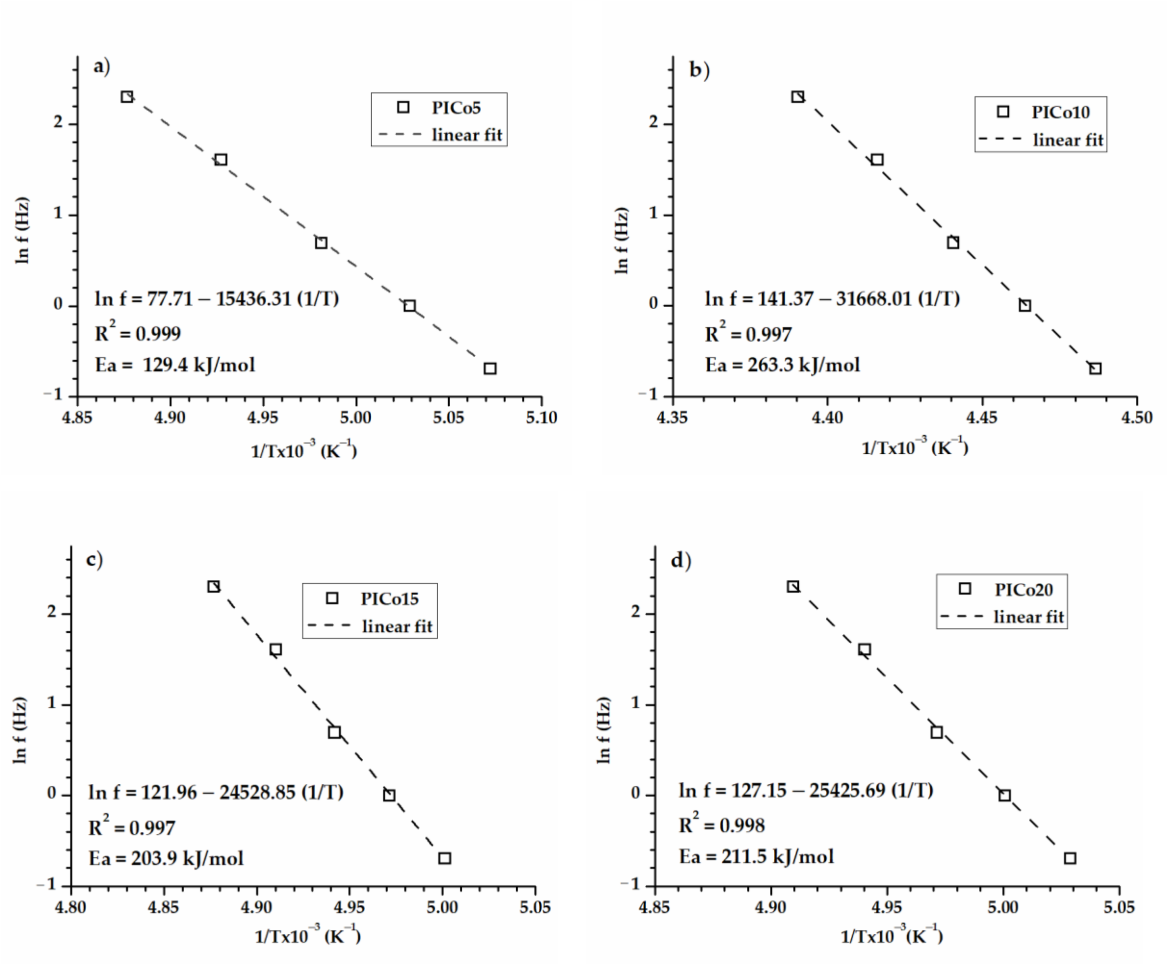

Figure S1. The Arrhenius plot of the γ_{Co} secondary relaxation for the samples PICo5, PICo10, PICo15 and PICo20 and the corresponding activation energy (Ea).

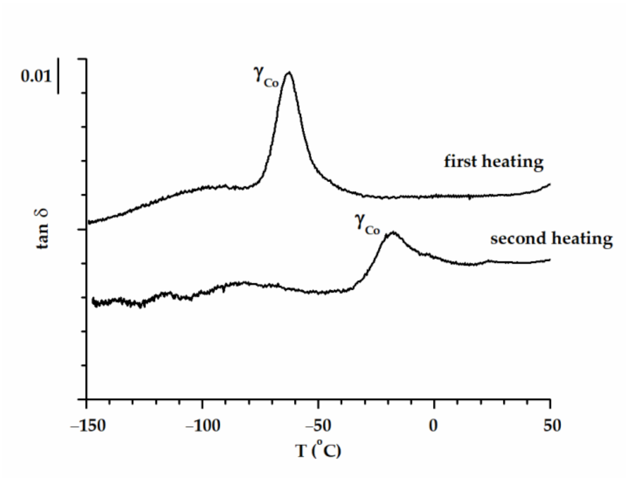

Figure S2. The γ_{Co} relaxation for the sample PICo15, during the first heating stage and the second heating stage. The isochronal experiment (1 Hz) was performed with 2 °C/min during all heating and cooling stages. The first heating stage was stopped at 125 °C and the sample was maintained 10 minutes at this temperature, before the cooling.
